# Reliability of a wellness inventory for use among adolescent females aged 12–14 years

**DOI:** 10.1186/1472-6874-14-87

**Published:** 2014-07-21

**Authors:** Jerome N Rachele, Thomas F Cuddihy, Tracy L Washington, Steven M McPhail

**Affiliations:** 1 School of Exercise and Nutrition Sciences and Institute of Health and Biomedical Innovation, Faculty of Health, Queensland University of Technology, Victoria Park Rd, 4059 Brisbane, Australia; 2 Civil Engineering and Built Environment School, Science and Engineering Faculty, Queensland University of Technology, Brisbane, Queensland, Australia; 3 School of Public Health and Social Work and Institute of Health and Biomedical Innovation, Faculty of Health, Queensland University of Technology, Queensland, Australia; 4 Centre for Functioning and Health Research, Queensland Department of Health, Queensland, Australia

**Keywords:** Wellness, Inventory, Reliability, Adolescent, Female, Test-retest

## Abstract

**Background:**

The wellness construct has application in a number of fields including education, healthcare and counseling, particularly with regard to female adolescents. The effective measurement of wellness in adolescents can assist researchers and practitioners in determining lifestyle behaviors in which they are lacking. Behavior change interventions can then be designed which directly aid in the promotion of these areas.

**Methods:**

The 5-Factor Wellness Inventory (designed to measure the Indivisible Self model of wellness) is a popular instrument for measuring the broad aspects of wellness amongst adolescents. The instrument comprises 97 items contributing to 17 subscales, five dimension scores, four context scores, total wellness score, and a life satisfaction index. This investigation evaluated the test-retest (intra-rater) reliability of the 5F-Wel instrument in repeated assessments (seven days apart) among adolescent females aged 12–14 years. Percentages of exact agreement for individual items, and the number of respondents who scored within ±5, ±7.5 and ±10 points for total wellness and the five summary dimension scores were calculated.

**Results:**

Overall, 46 (95.8%) participants responded with complete data and were included in the analysis. Item agreement ranged from 47.8% to 100% across the 97 items (median 69.9%, interquartile range 60.9%-73.9%). The percentage of respondents who scored within ±5, ±7.5 and ±10 points for total wellness at the re-assessment was 87.0%, 97.8% and 97.8% respectively. The percentage of respondents who scored within ±5, ±7.5 and ±10 for the domain scores at the reassessment ranged between 54.3-76.1%, 78.3-95.7% and 89.1-95.7% respectively across the five dimensions.

**Conclusions:**

These findings suggest there was considerable variation in agreement between the two assessments on some individual items. However, the total wellness score and the five dimension summary scores remained comparatively stable between assessments.

## Background

The concept of wellness is an important construct that offers a point of difference to other health-related concepts. Wellness has been described as a dynamic process maximizing an individual’s potential [1], and an active process through which the individual becomes aware of and makes choices toward a more successful existence [2]. Wellness can be conceptualized as a multi-dimensional holistic notion, focusing on the individual’s journey to being the best that they can be, within the environment where they are situated. Its focus on positive behaviors leading toward this outcome is an important point differentiating it from other constructs that may primarily focus on the prevention or treatment of disease or disability. Wellness aims to promote behaviors which facilitate the life journey and is about actions or processes rather than outcomes.

The wellness construct has application in a number of fields including education and healthcare, though most notably in the field of counseling, particular among adolescent females [3]. Wellness instruments may be used both as educative self-assessment tools to identify at-risk elements of an individual’s life, and as a tool to facilitate the counseling process. When compared to their male counterparts, adolescent females are more likely to: be bullied on school property and electronically, be forced to have sexual intercourse, feel sad or hopeless, seriously consider suicide, make a suicide plan, attempt suicide, have alcohol given to them, use an inhalant, not eat for ≥24 hours, and vomit or take laxatives to lose weight or to keep from gaining weight [4]. Consequently, the use of this tool may have particular relevance to adolescent females. Measuring the wellness of adolescents can assist researchers and practitioners in determining lifestyle behaviors in which adolescents are deficient. Behavior change interventions can then be designed which directly aid in the promotion of these areas. For example, Smith-Adcock et al. [3] examined the use of a group intervention counselling intertwined with wellness concepts (using the Wheel of Wellness model [5]) for adolescent females at risk of delinquency. Findings indicated that the wellness intervention helped the participants broaden their ideas about wellness and to set personal wellness goals.

Numerous theories and models have been created to represent wellness, all of which encompass a diverse range of lifestyle dimensions. One such model of wellness that has been developed, largely based on the psychology of Alfred Adler, is the Wheel of Wellness (WoW) [5], and the subsequent empirically-based Indivisible Self Model of Wellness (IS-Wel). Wellness assessment instruments derived from these theories allow researchers and practitioners to apply these concepts in a variety of contexts and settings.

The 5-Factor Wellness Inventory (5F-Wel) is a popular instrument for measuring the broad aspects of wellness among adolescents [6,7]. In combination with its predecessor, the Wellness Evaluation of Lifestyle [8], it has been an extensively used instrument in this population [3,9-14]. The 5F-Wel instrument is designed to measure the IS-Wel wellness model. This is an empirically-based model, developed from a factor analysis of Wellness Evaluation of Lifestyle data [15]. The IS-Wel is grounded in Adlerian counseling theory [16] that emphasizes the indivisibility of the self. This is what Adler defined as holism, and is based on a single, higher order wellness factor that includes all wellness components [6].

Although the 5F-Wel has been used among adolescents, there has been no empirical investigation regarding its reliability in this population. A lack of reliability of instruments for measuring wellness in adolescent populations has been highlighted as a concern in previous research [17]. Following on from this suggestion, Rachele, Cuddihy, Washington, & McPhail [18] conducted a test-retest reliability of the 5F-Wel instrument in adolescent males, finding favorable results. Prior studies involving the 5F-Wel have reported factor analysis for the five second-order factors (dimensions of self) [19], and internal consistency [20] of the instrument within adult populations. However, there have been no previous peer-reviewed investigations of test-retest reliability of the instrument amongst adult or adolescent female populations. It is important that reliability of the 5F-Wel is established among female adolescents to support its use in observing wellness in a cohort at a single time point, change over time or the effect of positive behavior-based interventions on wellness. The aim of this investigation was to evaluate the test-retest (intra-rater) reliability of the 5F-Wel instrument in repeated assessments made by adolescent females aged 12–14 years.

## Methods

### Instrument

The 5F-Wel T version (the 5F-Wel modified to a 6^th^ grade reading level) is a 97 item questionnaire which includes attitudinal and behavioral statements (e.g., “I eat a healthy diet”) that respondents rate their agreement with the statement using a 4-point Likert scale ranging from strongly agree (1) to strongly disagree (4) [6]. The instrument takes approximately 15 minutes to complete. The 97 questions are grouped to contribute to 17 subscales, four context scores and an overall life satisfaction index [6]. Mean item ratings for each subscale are computed and modified using a linear transformation to make the scales comparable, with each having a range from 25–100 [6]. The 17 subscales can be grouped into five dimensions of self (creative, coping, social, essential and physical) that comprise the main outcome variable, total wellness [6]. The creative self dimension includes the subscales thinking, emotions, control, work and positive humor. The coping self dimension includes the subscales leisure, stress management, self worth and realistic beliefs. The social self dimension includes the subscales friendship and love. The essential self dimension includes the subscales spirituality, gender identity, cultural identity and self-care. The physical self dimension contains the subscales exercise and nutrition. Detailed definitions of dimensions and subscales and information on theory development have previously been described [15], as have the methods for calculating instrument outcome scores [20]. When designing the methodological approach for this study, the investigators decided to follow the approach for calculating subscales and dimension scores described in the instrument manual [20]. This involved the numerical summation and averaging of ordinal data during the calculation of summary scores. While the investigators had some concerns with this approach, it was considered important to follow the instrument manual instructions to ensure that the findings of this study are meaningful to instrument users (who would likely use these same instructions).

### Study design and participants

This intra-rater reliability investigation required respondents to self-complete the 5F-Wel on two separate occasions (test-retest), with seven days between assessments. A convenience sampling approach was used (n = 48) with participants required to be female, and aged between 12 and 14 years to be eligible for participation in this study. Participants were from an all-female secondary school with Catholic affiliation in a metropolitan area of Brisbane, Australia.

### Procedure

Participants completed their initial 5F-Wel report during class time, at their school. Participants then completed the 5F-Wel seven days later in the same scheduled class. A seven day period was chosen to decrease the chance of participants recalling their response from the previous administration while also minimizing the chance of substantial life changes between assessments [21]. Consent was obtained from both the participants, and a parent or guardian. This study was approved by the Human Research Ethics Committee of the Queensland University of Technology.

### Data analysis

Data analysis was completed using StataIC version 11 [22]. To investigate the stability in individual item responses between the two assessments, the percentage exact agreement was calculated for each of the 97 items of the 5F-Wel. While the individual item level stability between assessments has relevance to the instrument reliability, the investigators considered it likely that respondents in this adolescent sample may not necessarily provide exactly the same responses from week to week for many individual items; even if item meaning were consistently interpreted in the same way. Therefore, it was considered perhaps more pertinent to examine the relative stability of the main summary variable derived from the instrument ‘total wellness’ and the five summary dimension scores of self (creative, coping, social, essential and physical). The investigators considered that while individual item responses may have some natural variation from week to week within individuals, it was less likely that their total wellness (or the five aforementioned dimensions of wellness) would genuinely change over a seven day time-frame. Therefore, to examine the stability of total wellness scores and the five dimensions of self between the two assessments, the number of respondents who scored ±5, ±7.5 and ±10 points were calculated for each of these variables.

## Results

Overall, 46 (95.8%) responded with complete data and were included in the analysis. One participant had incomplete responses to the first assessment and another was absent from school for the second assessment. Both participants were excluded from all analyses. The mean age and standard deviation (SD) of participants was 13.26 (0.57) years.

The percentage exact agreement between the two assessments for each of the 97 items of the 5F-Wel are displayed in Table 1. There was substantial variation in agreement levels between the two assessments across the 97 items; with the total range from 47.8% to 100%. The median (and interquartile range) percentage agreement across the 97 items was 69.6% (60.9% to 73.9%). Only one item (relating to tobacco usage), had 100% agreement between the two assessments.

**Table 1:** Percentage agreement each of the 97 items on the 5F-Wel

**Item**	**Percentage agreement**	**Item**	**Percentage agreement**	**Item**	**Percentage agreement**	**Item**	**Percentage agreement**
1	69.6	26	71.7	51	50.0	76	76.1
2	65.2	27	67.4	52	67.4	77	80.4
3	89.1	28	65.2	53	73.9	78	82.6
4	73.8	29	65.2	54	52.2	79	73.9
5	71.8	30	71.7	55	63.0	80	67.4
6	71.7	31	78.3	56	54.4	81	69.6
7	63.0	32	73.9	57	60.9	82	69.6
8	95.7	33	71.7	58	73.9	83	71.7
9	76.1	34	56.5	59	60.9	84	76.1
10	73.9	35	56.5	60	78.3	85	67.4
11	76.1	36	80.4	61	78.3	86	71.7
12	60.9	37	65.2	62	54.4	87	67.4
13	65.2	38	67.4	63	78.3	88	60.9
14	54.4	39	58.7	64	97.8	89	71.8
15	100.0	40	69.6	65	71.7	90	67.4
16	60.9	41	76.1	66	80.4	91	78.3
17	58.7	42	73.9	67	67.4	92	76.1
18	63.0	43	73.9	68	76.1	93	65.2
19	91.3	44	65.2	69	56.5	94	58.7
20	67.4	45	47.8	70	65.2	95	71.7
21	73.9	46	56.5	71	73.9	96	54.4
22	56.5	47	82.6	72	73.9	97	47.8
23	67.4	48	60.9	73	69.6		
24	52.2	49	76.1	74	60.9		
25	71.7	50	54.4	75	69.6		

Absolute differences between assessments for total wellness (scale range 25 to 100) was 87.0% for within 5 points, 97.8% for within 7.5 points, and 97.8% for within 10 points. Absolute differences between assessments for each of the five dimension scores ranged from 54.3% (physical self) to 76.1% (social self) for within 5 points, 78.3% (physical self) to 95.7% (essential self) for within 7.5 points, and 89.1% (social self) to 95.7% (creative self and essential self) for within 10 points.

## Discussion

This study was the first investigation of reliability for the 5F-Wel instrument amongst female adolescents and the second in an adolescent population. The investigation has revealed some interesting findings regarding the relative stability of responses to the 5F-Wel instrument among adolescent females. Specifically, the stability of responses between assessments conducted seven days apart had substantial variation across the individual items (Table 1). Despite this, the total wellness score and each of the five dimension scores displayed relative stability; particularly at the 7.5 and 10 point absolute difference margins of error (Table 2). This suggests that while adolescents in the sample may have changed their responses to some of the individual items from week to week, the variables likely to be used in research and clinical practice (total wellness and five dimension scores) remained quite stable between assessments. These findings provide some evidence to support the intra-rater reliability of the instrument when administered among adolescent females, particularly when using outcome scores from the five dimensions and total wellness. However, it also suggests caution when considering changes between longitudinal assessments at an individual item level, given that some respondents may alter their responses to individual items from one week to the next.

**Table 2:** **The percentage of respondents who scored within** ±**5**, ±**7.5 and** ±**10 points for each of the 5 dimensions and total wellness of the 5F-Wel**

	**Within absolute difference**		
**Measure**	**5 points (%)**	**7.5 points (%)**	**10 points (%)**
Creative Self	71.7	87.0	95.7
Coping Self	71.7	89.1	91.3
Social Self	76.1	89.1	89.1
Essential Self	69.8	95.7	95.7
Physical Self	54.3	78.3	91.3
**Total Wellness**	**87.0**	**97.8**	**97.8**

The 5F-Wel dimension which seemed to demonstrate the least stability between the two assessment points was physical self. It was not surprising that this dimension, which includes dietary and exercise behaviors, demonstrated the least stability between assessments among this sample of adolescent girls. It has previously been suggested that responses to dietary behavior questions may be subject to significant variation from week to week among this age group [23]. However, it was also noteworthy that this dimension still had frequent agreement at the ±10 point level between the two assessments (91.3%). The dimension that demonstrated the most stability between the two assessments was social self. Social self is described as consisting of “friendship” and “love”, where the mainstay of social support is within families [20]. It is likely that family circumstances are unlikely to change from week to week, and it is therefore unsurprising that this dimension showed the greatest level of agreement between assessments.

Reliable measures of wellness are important for observing the effect of interventions on wellness, to observe wellness in a sample cohort at a single time point or changes that may occur over time. This study has provided some foundational empirical evidence regarding the reliability of this instrument (specifically, the stability in interpretation of the items, dimensions and total wellness variables) among adolescent females. This evidence is important to inform multi-dimensional wellness instrument selection for observational studies and intervention evaluation targeted at adolescent females [18]. Findings from this research support the use of the 5F-Wel dimensions and total wellness score for this purpose.

The level of reliability reported in this investigation are comparable with other self-reported instruments amongst adolescents such as the Adolescent Physical Activity Recall Questionnaire (67 – 83%) [24] and World Health Organisation, Health Behavior in School-Aged Children food frequency questionnaire (37-87%) [23]. Furthermore, the level of reliability observed in this investigation is also comparable to that observed when similar wellness instruments have been evaluated amongst adult populations [25,26].

A study of this nature will always face two potential risks [21]. First, there is the innate risk that a participant may have anticipated the purpose of the study, recalled their original answer and responded in the same way when completing the questionnaire for the second time. The second is the risk that a participant’s life situation or attitudes to the assessment statements may have measurably changed between the two assessment points. We believe that this study was more at risk of the second limitation than the first as a seven day period was allowed between assessments. This, combined with the number of items (n = 97) that a respondent would have had to remember correctly gave some protection against the memory-recall limitation. By doing so however, our results were likely to be more conservative than what could be expected in real life. Hence, given the nature of our design, we argue that the results of this investigation provide evidence to support the use of the 5F-Wel among female adolescent populations. This may comprise a number of settings including research environments, schools or community centers, and with practitioners such as social workers, school-based nurses and counselors.

There are also several factors that may limit the extrapolation and transferability of findings from this study. A convenience sampling approach was used to recruit a relatively small sample from one geographical region. Data collection was undertaken in a Catholic private school in a developed nation where adolescents are likely to have a high level of literacy. It is unlikely that socioeconomically or educationally disadvantaged adolescents were represented in this sample and may not have responded in the same way as participants in this study. While this investigation provides important foundational empirical evidence for use of the 5F-Wel instrument, there are several related research priorities. In addition to investigating the reliability of the 5F-Wel amongst socioeconomically or educationally disadvantaged adolescents, the reliability of the instrument across possible alternative modes of administration should also be a priority for future research. In this study the 5F-Wel was administered as a self-completed paper-based questionnaire. Two alternative modes of administration worthy of investigation amongst adolescents include computer administration (such as via a web-based survey platform) and telephone administration. These two alternative modes of administration, if reliable, may facilitate 5F-Wel completion in professional and research contexts. Web-based administration may increase the feasibility of large scale investigations and offer a convenient alternative for computer savvy adolescents. Telephone administration may improve response rates for investigations where participants have not completed and returned the paper based version. However, for telephone reliability to be established, it may be prudent to first investigate whether the 5F-Wel questions elicit the same response when self-completed versus interviewer administered. It is foreseeable that an adolescent may not provide the same responses to an interviewer than when self-completing the instrument in relative privacy. Any discrepancy observed between interviewer administration and self-completion of the instrument may also influence an inter-mode reliability study investigating telephone administration of the 5F-Wel.

## Conclusion

This study has been the first to investigate the reliability of the 5F-Wel instrument amongst adolescent females. The findings suggested there was considerable variation in agreement between the two assessments on some individual items. However, the total wellness score and the five dimension summary scores remained comparatively stable between assessments; providing evidence in support of the intra-rater reliability of the total wellness and dimension scores generated from the 5F-Wel instrument.

## Competing interests

The authors declare that they have no competing interests.

## Authors’ contributions

All authors contributed to the conception and design of the manuscript. JNR, TFC and TLW contributed to the acquisition of data, while JNR and SMM contributed to the analysis and interpretation of data. All authors contributed to the drafting and revising the article critically for important intellectual content, and gave final approval for the version to be submitted and any revised version.

## Pre-publication history

The pre-publication history for this paper can be accessed here:

http://www.biomedcentral.com/1472-6874/14/87/prepub
